# Governing healthcare: the uses and limits of governmentality in the National Health Service in England

**DOI:** 10.1080/13648470.2023.2242280

**Published:** 2023-10-02

**Authors:** Lorelei Jones

**Affiliations:** Medical and Health Sciences, Bangor University, Bangor, UK

**Keywords:** Governmentality, healthcare, policy, expertise, professionals, ethnography, NHS, England

## Abstract

Using examples from the National Health Service in England, this paper illustrates key features of contemporary healthcare governance: the way decisions are hidden in places that are ‘in between’ and ‘out of reach’; the enrolment of doctors in governing; and the important role played by ‘boring things’, such as power point slides, flow charts, and forms. The essay shows how anthropological proximity and perspectives can extend and deepen understanding of contemporary political power. It does this firstly by showing the importance of agency in the operation of governmentality, and secondly by illuminating the limits of governmentality. The different elements of governing assemblages, such as global management experts, medical leaders, forms of knowledge and analytical technologies, are brought together through the strategic act of framing. Frames are contested and resisted, requiring more visible forms of control.

In this essay I illustrate key features of contemporary healthcare governance, drawing on fieldwork in the National Health Service (NHS) in England between 2007 and 2022. The selection of ethnographic moments that follow are used to show how decisions are hidden in places that are ‘in between’ and ‘out of reach’; how doctors are enrolled in governing; and how ‘boring things’ (Star 1999) such as power point slides, flow charts, and forms, shape what people do. I explore how these tactics and technologies are incorporated into the political strategies of powerful government actors alongside formal authority, and how they are contested and resisted. The essay shows the unique contribution of anthropology to understanding political power, by foregrounding everyday practices, and the way people use cultural resources to accomplish activities.

For anthropologists, public policy is a multi-sited cultural phenomenon (Marcus 1995). It has a temporal and geographical character that invites creative conceptions of the ‘field’ to transverse locales and domains. Over the last fifteen years I have generated a multi-sited research strategy to study healthcare policy by, variously, ‘following the policy’, ‘following the knowledge’ and ‘following the leader’, through relations between actors, institutions, and discourses, across time and space (Wright and Reinhold 2011).

‘Following the policy’ has meant tracking how a healthcare policy is articulated and enacted at different levels (global, national, regional, local), the dilemmas that policy generates for people and how they respond, and how these interactions shape the material effects of policy (Jones 2018; Jones and Exworthy 2015). I have also ‘followed’ different practices and agents that are assembled by policies, such as management knowledge (2018) and medical leaders (2021), to see where they go, and what they do. This approach has enabled me to capture and connect the different actors, arenas, and practices of healthcare governance. For example, I have explored how a national policy for more ‘self-care’ brought together think tanks, local healthcare managers, management knowledge, and clinical staff, to shape the conduct of citizens (Jones 2018). Anthropological attention to the strategies of local healthcare managers revealed how they went about attempting to change clinical practices, using models, tools and techniques circulated by think tanks. Anthropological proximity also meant I could see how clinical staff resisted new subjectivities when these competed with alternative sources of meaning and foundations for action, such as professional identities.

My approach draws on Foucault’s theories of modern power. These ‘analytics’, as he refers to them, focus on the nature and workings of power, underscoring the many different forms of power involved in governing, and illuminating those that are difficult to see, such as the power that is immanent in knowledge. It is this conjunction of knowledge and power that he refers to as discourse (1990, 100). Discourses are embodied in speech and writing, but also in the social practices of everyday life, and even the physical layout of social institutions, such as hospitals. Both an instrument and an effect of power, discourses constitute political objects (such as ‘care’) and subjects (such as ‘doctors’ and ‘patients’) positioning them in relations of power. They are ‘tactical elements’ operating in force relations, and there can be different, and even contradictory, discourses within the same strategy (1990, 102). As an instrument of power discourses are effective because they are difficult to see, but they can also be ‘a hindrance, a stumbling block, a point of resistance’ (1990, 101).

Governmentality is an indirect form of power that works through an ‘ensemble’ of different elements, including institutions, procedures, tactics, analyses, and calculations (Foucault, Burchell, and Gordon 1991, 102). Miller and Rose (1993) apply this perspective to public policy, highlighting the role of professionals in governing assemblages. They argue that ‘action at a distance’ is accomplished through professionals, such as doctors, whose authoritative norms, calculative technologies, and forms of evaluation are translated into the values, decisions, and judgements of citizens in their professional and personal capacities (Miller and Rose 1993, 92). Professionals make it possible for self-regulation to operate in a way that minimises the need for direct political intervention, acting as

Powerful translation devices between “authorities” and “individuals” shaping conduct not through compulsion but through the power of truth, the potency of rationality, and the alluring promises of effectivity. (Miller and Rose 1993, 93)

Ferlie and McGivern (2014) argue that governmentality is a useful theoretical resource for understanding indirect forms of control in ‘post bureaucratic’ contexts, such as the NHS. They identify two important elements of healthcare governance in England – local governing agents in the form of doctors who have taken on management roles, and a ‘health economics/clinical outcomes knowledge hybrid’ as the underpinning rationality. Their empirical setting is a sexual health network. The clinical network is an organisational form based on horizontal collaboration between different organisations, rather than control through vertical line management. Ferlie and McGivern describe ‘energetic’ local medical leaders who have internalised national policy objectives for the standardisation of clinical services and see themselves as powerful change agents, able to successfully implement changes to clinical services through their ability to ‘engage’ clinical colleagues.

The anthropology of policy contributes to an understanding of governmentality by showing how policy aligns different elements of governing assemblages. Shore and Wright, for example, state that:

It is precisely the way that policy creates links between agents, institutions, technologies and discourses and brings all these diverse elements into alignment that makes it analytically productive. (Shore, Wright, and Però 2011, 11)

I develop this perspective by showing how the knowledge and activities of doctors, and their perceived neutrality and social authority, are mobilised through the agential act of ‘framing’. The concept of interpretive frames stems from the work of Goffman (1974) who defined frames as organizing principles that govern the meaning we assign to social events (1974, 10). Rein and Schön apply the concept to the study of public policy, using the term to refer to ‘a perspective from which an amorphous, ill-defined, problematic situation can be made sense of and acted on’ (Rein and Schön 1993, 146). Policy actors have different frames that lead them to see things differently and support different courses of action concerning ‘what is to be done, by whom, and how to do it’ (1993, 147). The concept of framing allows exploration of the multiple ways that power operates in policy practices, both with and without intention. Frames can be taken for granted, so that ‘we are often unaware of their role in organizing our perceptions, thoughts, and actions’ (Rein and Schön 1993, 151), but can also be an intentional political strategy. Indeed, Rhein and Schön suggest that ‘reframing’ may be a way out of a situation where conflicting frames have produced policy paralysis.

Frames ‘work’ in different ways, as rhetorical devices that extract legitimacy from cultural resources, such as medical science, and democratic participation, and as discursive strategies that shape how the problem is understood, which solutions are considered (and which are not), and who is included in decision making (Jones and Exworthy 2015).

While it may seem that systems of power are made up of ‘boxes within boxes within boxes’, Abu-Lughod (1990) suggests this is the wrong image: ‘A better image might be fields of overlapping and intersecting forms of subjection…’. This essay is a necessarily partial account of this complex interplay, focusing on the way governmentality is used by powerful actors alongside sovereign power, and how both forms of power are contested and resisted.

## Research setting

The public healthcare system in England is run directly by the UK government. It is funded through general taxation and is largely free at the point of use to citizens. Established by a Labour government as part of the post war ‘welfare state’, its founding principle is that care should be based on need, and not ability to pay. As such the NHS is politically visible and often subject to large-scale re-organisation. The residue is a complex and fluid patchwork of organisations and lines of accountability. Localities vary in terms of geography, organisational context, and political cultures, however, a notion of ‘the NHS’, in the singular, persists, indexing values such as fairness and security, and attracting public affection.

In general, the local healthcare field is made up of operational managers, clinicians and other healthcare staff who are employed, on the most part, by public sector organisations that either commission (plan, purchase and manage) or provide health services to the local population. For much of the time that I have been doing fieldwork, local managers ‘looked up’ to more senior managers in a regional tier of the NHS (henceforth ‘regional offices’). The formal role and functions of regional offices, and how are enacted, has varied, and shifted over time, but they were often viewed as the ‘managerial arm’ of the National Department of Health (Edwards and Buckingham 2020). And although individual management style varied, the following extract from an interview account illustrates the coercive nature of hierarchical control, at least in some localities in England:

[The regional office CEO] said “Look in this drawer. I have undated letters of resignations from every chair and every chief executive in my patch and that’s my policy because if they ever stand out of line then I will just date them and sign them”. (Edwards and Buckingham 2020, 18)

Recently Pushkar (2023) has shown how coercive pressure exerted through hierarchy constrains the moral reasoning and political action of local NHS managers. Pushkar found that local managers did imagine alternative possibilities but kept quiet because they were afraid of losing their jobs. They trumpeted the benefits of changes to clinical services and minimised the risks ‘*because they were made to’* (Pushkar 2023, 133).

The period of ethnographic fieldwork covers three different national governments: a Labour government (1997-2010); a Conservative-Liberal Democrat Coalition (2010-2015); and a Conservative government (2015-present). All three governments have pursued a neoliberal agenda in terms of extending market relations and promoting personal responsibility for health (Larner 2003; Miller and Rose 2008). Since 2010 funding to the NHS has been restricted, and a key objective of government has been major efficiency savings.

During my time in the field, I have seen healthcare policies ‘zig zag’ between different modes of governance (hierarchy, markets, and networks). Rather than replace one another, these policies have accumulated, resulting in multiple, conflicting, policies and a complex discursive field (Jones 2017). I have previously argued that this complexity is the result of the combination of neoliberal regimes, committed to decentred forms of coordination and control, and what Lyn refers to as the ‘persistence of hierarchy’ in contexts, such as the NHS, ‘where citizens and their representatives will, in one way or another, sooner or later, insist on accountability on the part of those who act in their name using resources appropriated from them’ (Lynn 2011, 231). In this paper I consider the way indirect, and less visible, forms of control are used by powerful government actors, alongside hierarchical power.

## A note on methods

During fieldwork I talked to policy makers in the Department of Health (now the Department of Health and Social Care) and other national organisations. I also talked to regional strategic managers, local operational managers, clinicians, and residents. I observed planning meetings, visited offices, hospitals, community centres, and educational facilities, and read national policy documents, regional and local planning documents, and local reports and meetings papers. Fieldwork has involved over 130 different organisations (NHS, Local Authority, private and voluntary sector) in every region of England.

For much of this time I was working as a research fellow on a series of studies funded by the government through the NHS Service Delivery and Organisation programme, the Department of Health Policy Research Programme, and the National Institute for Health Research. These included national evaluations of healthcare policies, and research on hospital governance and quality improvement. This meant that I was both observing and participating in governing practices. While this gave me ample opportunity for ‘thinking in the world’ (Ingold 2014), it meant that I was thinking/writing for a policy and practice community, while at the same time completing a PhD in Anthropology and thinking/writing for a critical social science community. In addition to ethical issues (see Jones 2018), a key struggle has been ‘making the familiar strange’ when faced daily with the seductive technical discourses of Health Services Research, management knowledge, and rational policy analysis. Immersing myself in social science literature, conferences, and conversations has helped me find ‘a different language’ (Ball 1997), critical distance, and imagination. There have also been instances of productive dialogue between the two communities, as when I have written a paper for a social science journal, and then translated it, to distil insights for a policy and practice audience. But, from my experience, this is possible only *after* the long, painstaking processes of developing an anthropological account.

## Hidden decisions

The offices of the Strategic Health Authority were hard to find, hidden in a nondescript building, on a nondescript street, in a leafy suburb in one of the home counties. This was in Spring 2007 and early in my fieldwork on a study that would form the basis of my doctoral research on hospital planning in England. The Strategic Health Authority was, at that time, the regional office of the National Health Service, one of ten in England that aligned geographically with the old government office regions. Their role was to develop strategy, implement national policy, and manage performance of NHS organisations. Unlike the District Health Authorities before them, Strategic Health authorities did not have a board, but instead reported directly to the national Department of Health. I didn’t know any of this at the time. I didn’t know what the Strategic Health Authority was, and I didn’t know where it was.

Hospital planning, like all decisions about the distribution of collective goods, is inherently political. In England plans to close or downgrade hospitals are often highly contested, involving not just conflicts of interest, but conflicts in meaning. A central conflict is between the instrumental rationality of healthcare management and the many social, emotional, and symbolic attachments that hospitals hold for local communities. A recent study of community hospitals in England show how they are embedded in local communities, employing local people, and contributing to social and emotional dimensions of care. Patients felt less anxious in familiar surroundings, ‘close to home’, and easily visited by family and friends. They also *felt* safe because they were known to staff (Davidson et al. 2022). In England, hospitals are also symbolic of political values, such as communality, and of an ideological commitment to a national, comprehensive health service (Brown 2003). As Glasby, Smith, and Dickinson (2006) observe ‘hospitals are much more than just buildings where health care is delivered, but the physical incarnation of the NHS and its values within a particular locality’.

Successive national governments in England have had a policy to reduce the number of acute hospital beds, by downgrading or closing hospitals. But like the regional office, it is hard to find. I heard it once, in 2012, in a glass-walled meeting room in the London offices of the Department of Health (as it was then). It was just me and a civil servant and we were discussing a policy evaluation that I was working on as a member of an academic team, when he said: ‘our priority is to take capacity out of the acute sector’. But as a policy that is too sensitive to be articulated (Yanow 1987), it is difficult to find this written down in published policy documents. Instead, for much of the last fifty years, responsibility for hospital planning has been devolved to regional offices. Although, as Stewart et al. (2020) have shown, the decisions of regional offices are often in response to, and under the close scrutiny of, national-level policy actors.

Sitting between the more familiar levels of national and local government, the regional level of government is almost completely unknown to the people affected by decisions – the pubic, patients, and front-line staff. It is also largely unscrutinised by journalists, local politicians, and academics. In the applied field of Health Services Research, the existence and workings of the regional tier is further obscured by the adoption of publishing conventions from biomedical science. In the passive sentence constructions of academic papers, programmes of large-scale organisational change that affect local organisations and clinicians simply exist, without subjects, so it is impossible to work out ‘who’, exactly, is behind them.

Devolving decision-making to regional administrators is a long-standing political technique used to buffer central government actors from criticism and immobilise opposition (Edelman 1975). It is a key form of ‘depoliticisation’ (Burnham 2001). Depolicitisation is the discursive denial of the inherent contestability of the provision of collective goods (Hay 2014). It has become embedded in the NHS, and more widely, through the rise and spread of managerialism (Hay 2014), so that Flinders and Wood (2014) describe it as ‘the dominant mode of statecraft of the twenty first century’. From a Foucauldian perspective, depoliticization is not removing politics but hiding it. The effect is not less politics but less visible politics (Foster, Kerr, and Byrne 2014). Political control is enhanced because decision making is hidden from view.

As a political tactic, *devolution* of decisions is often used alongside *delegation* to experts (Rose and Miller 1992). When I first began fieldwork in Spring 2007 the regional strategy for hospital services had been developed by a private firm of management consultants in response to a financial deficit. The management consultants recommended centralising hospital services onto fewer, larger sites (what they referred to as ‘reconfiguration’). An ‘off the shelf’ management solution (Ferlie 2016), it is a standardised model that continues to be sold to healthcare clients world-wide.

Global private sector management consultants are an important link in contemporary governing assemblages. A report from the international firm McKinsey to the Department of Health (McKinsey 2009) is revealing, showing the comprehensive influence of management knowledge, and global management experts, on Health Policy in the English NHS, supplying the priorities (major efficiency gains); individual policies (e.g. reconfiguration of local health economies); and the implementation strategy (e.g. ‘drive through the regional offices’, ‘remove barriers, such as resistance to reconfiguration’, ‘use a compelling story’). In subsequent years all the recommendations were adopted by the Department of Health. Management consultants have also advised most regional offices on how efficiency savings could be produced (McGivern et al. 2018). In my fieldwork they were ever present ‘shadow elites’ (Wedel 2009) that influenced decision-making in a way that had significant implications for the distribution of collective goods, but in the guise of providing unproblematic technical solutions.

Rhodes (2013) has observed that one artefact of managerialism is the ‘slide pack’ as the dominant form of communicating analysis. McGivern et al. (2018) describe it as a discursive and hegemonic boundary object, imposing an expert, abstract, and technical understanding of healthcare services that serves the interests of management consultants. In my case the report from the management consultants to the regional office took the form of a slide pack containing 311 power point slides. Some of these are shown in Figure 1.

Figure 1.Management consultancy analysis for the Strategic Health Authority.

**Figure:**
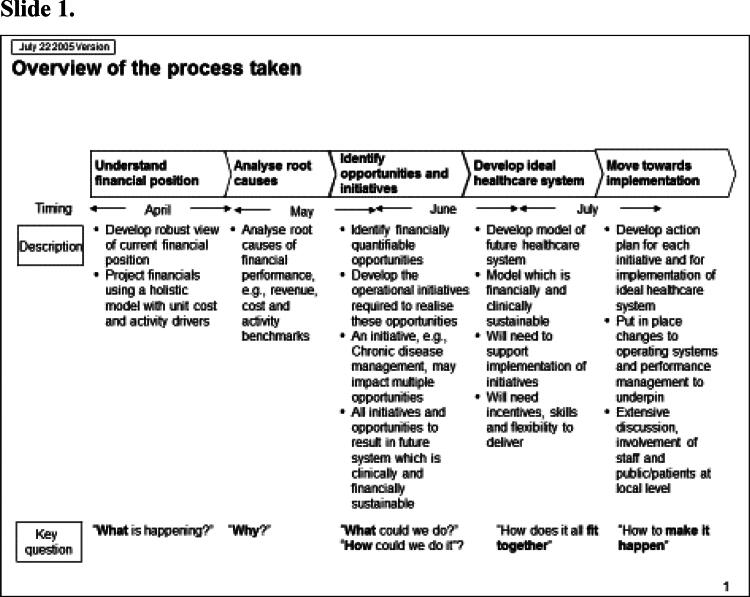

**Figure:**
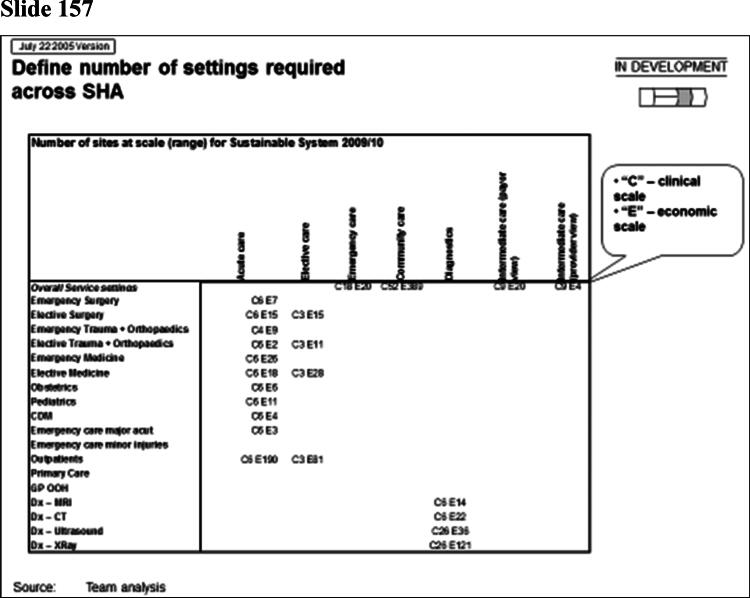

**Figure:**
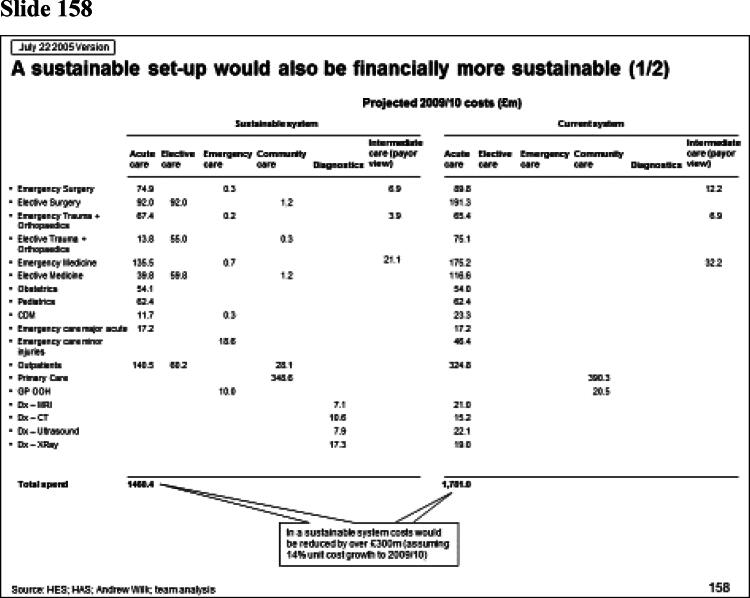

**Figure:**
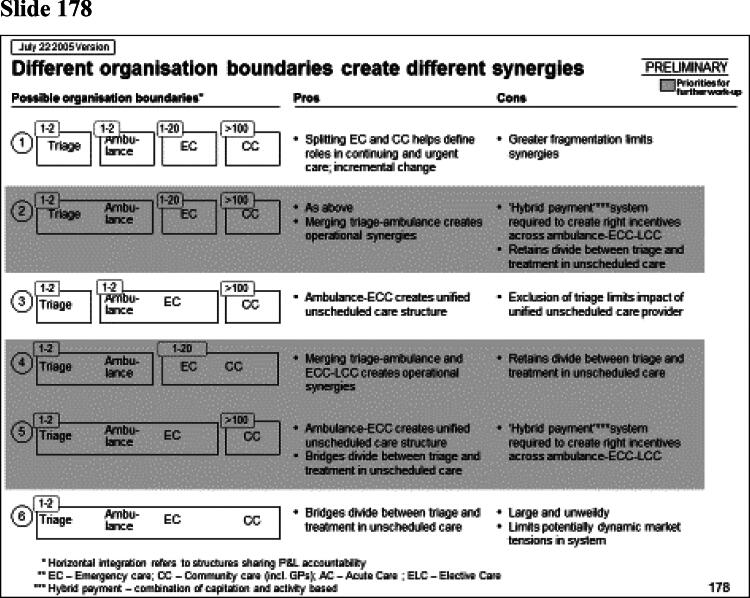

The analysis arranged the various component services (accident and emergency, surgery etc) into the ‘ideal’ healthcare system, depicted in a series of flow charts. The first slide gives an overview of the process. It depicts a linear, rational process, where the objective to ‘develop ideal healthcare system’ is clearly identified and depicted as a response to the financial position. Note the location of the task of ‘involvement of staff and public/patients at local level’ under ‘implementation’. In other words, the process as depicted proposes to involve staff at the time of implementation – not before, or during, decision-making. Indeed, the decision was taken by a regional government agency on the advice of a private firm of management consultants.

Slide number 157 illustrates the way analysis is confined to the objectives of efficiency and service effectiveness. The slide presents the ideal number of departments in the region for each specialty. It gives two numbers, the ideal number ‘economically’ (E) and ‘clinically’ (C). The latter is derived from the recommendations of the national Royal Colleges (national bodies representing the different medical specialties) on the size of catchment population for each specialty department. The way the catchment population sizes are calculated are opaque, based on expert opinion from committees (Jones and Exworthy 2015). So, for example, the recommended number of departments for obstetrics, is ‘6’. Slide 158 then goes on to show that centralizing hospital services in the region would produce cost saving of £300 m. What is notable about the analysis is the way hospital services are abstracted from their social and cultural context. In slide 178 the boundaries of organisations have, literally, been redrawn. These flow charts might be seen as an inscription practice that transforms social reality, making it amenable to action (Rose and Miller 1992). The simplification involved in this process removes hospital services from their social and ideational settings.

## Doctors and governing

My fieldwork over the next two years was spent in one of several localities that made up the region. ‘The Shire’ is a large county is southeast England. It is mostly rural with a couple of large towns and some urban areas on the edge of greater London. It has 5 hospitals. At the time I was doing fieldwork regulators had expressed concerns about the financial position of all but one of these. March and Olsen note that when it comes to observing real-world decision-making and organisational change ‘it is difficult to describe a decision, problem solution or innovation with precision, to say when it was adopted and to treat the process as having an ending’ (1989, 63). The Shire had a long and complicated history of hospital planning. While the general principle of rationalisation had remained constant, the details, the constituent organisations, and the rationale for change had all changed over time and continued to change while I was in the field. Over the two years of fieldwork the plans had involved, at different times, closing a hospital; closing an accident and emergency department; closing a maternity unit; the regional centralisation of all acute services; and merging different hospitals.

On my first day in the field, it was immediately apparent that the plans were controversial. At the entrance of Shire General Hospital hung a large banner which read ‘Save our Shire General’. The ‘Save the Shire’ campaign involved local politicians, the local newspaper, local charities, hospital staff and members of the public. The campaign had arranged a petition, public meetings, and rallies and used creative forms of rhetoric, such as a candle-lit vigil outside the UK Parliament in London.

By the summer of 2007 the local plans for hospitals had been reframed, from a financial to a clinical rationale (Jones and Exworthy 2015). In planning meetings, documents, and public engagement events, the plans were repeatedly said to be ‘based on the evidence’ and ‘necessary for clinical safety’. As one local manager put it during a board meeting held in public, ‘this is what the doctors have told us we have to do’.

The reframing of the plans in the Shire mirrored developments in national healthcare policy. A key tactic was the mobilisation of medical elites. My ethnographic research supports Waring’s (2014) suggestion that doctors are co-opted into management and leadership roles in the interests of national policy makers. At the national level medical elites were appointed in a policy-making role alongside the minister. This was remarkable because, traditionally, it was civil servants who worked with the minister on policy, and in previous cases, such as the introduction of market-based reforms, the profession had been excluded from policy development. It is notably that it was in the controversial field of hospital planning that they were now included. The appointments were known as ‘tsars’ and were made for each specialty area (such as obstetrics and gynaecology, paediatrics, accident and emergency etc). In each case the ‘tsar’ published a standardised model of care (the same model as promoted by the international firm of management consultants), based on the centralisation of clinical services. Each model was known as ‘the clinical case for change’.

The national Department of Health also issued guidance to the regional offices on implementation strategies. As noted earlier, at this time it was the role of the regional offices to ensure that national policy was incorporated into local plans. The guidance issued to regional offices recommended ‘involving clinical staff at every stage of the process, from developing proposals and the case for change to implementation’:

Where clinical leaders genuinely develop and support proposals, they play a vital role in building public and patient confidence. In the best examples, medical directors have written forewords to consultation documents, clinicians have supported proposals at public meetings, articles have been written by the heads of relevant clinical disciplines and letters to correct local media stories have been sent from GPs. (Carruthers 2007, 6)

Many of these recommendations were adopted by local managers in the Shire. One way managers had reframed the plans was through a ‘co-design’ workshop with local doctors. The workshop was facilitated by the same firm of management consultants who had developed the regional strategy and held over two days with doctors from the local hospitals. The intention was to give the impression that the plans had emanated from doctors, rather than from a private firm of management consultants. This strategic use of medical expertise is apparent when ‘front stage’ planning talk is compared to ‘back stage’ planning talk (Degeling 1996). An example of ‘front stage’ talk is the report from the co-design workshop, written by the management consultants:

[The co-design workshop] enabled clinical leaders from across the three Trusts and from primary care to come together and use their experience and judgement to help guide the future investment and disinvestment decisions being faced by [the local] health economy. The clinical leaders worked in three groups – one for each clinical area. They were asked to use their expert opinion and judgement to develop sustainable service models that showed the levels of care and appropriate settings for them. They then considered these clinical models and discussed them with regard to options for service reconfiguration.

In contrast, when I spoke to the local doctors who had participated in the workshop, they expressed resentment at what they saw to be the superficial and strategic nature of their involvement:

it was led by [firm of management consultants] and they tried to lead us down a particular discussion line which we successfully ignored the first day, but they then presented a set of conclusions the following day which were completely unrepresentative of what we’d talked about on the first day, I mean I commented “I might as well not have been there” and finally they produced some sort of last-minute data which we hadn’t seen before the meeting and this sort of thing, it was all rather unpleasant and then we went away a bit disappointed in the process and when we saw the sort of draft, minutes if you like, of the meeting, it was a travesty, it bore no resemblance to the discussion we’d had and we were put under enormous amount of pressure as a Trust to just sign up and say “yes that’s what we discussed” and there were several [doctors] here who’d been involved, were very unhappy about that, we felt we’d been misquoted and we weren’t happy with the draft but the pressure on us to sort of accept it was quite large. (Senior hospital doctor, Shire General)

Doctors working in senior management roles in the Shire were also enrolled to ‘sell’ the plans in public forums. For example, the medical director from one of the hospitals was often the ‘front man’ for the plans during public engagement events (Jones and Exworthy 2015).

Anthropological attention to governing practices allowed me to capture not just how cultural resources, such as medical expertise, were mobilised by agents at different levels of government, but also the responses from other social groups, and the real-world effects. As a political technology the framing was effective in the sense that local managers were able to avoid public consultation. Local councillors accepted the representation of the plans as ‘clinical best practice’ that did not require democratic scrutiny (Jones and Exworthy 2015). However, as rhetoric, it was unsuccessful in that local staff and members of the local community were unconvinced by the ‘the clinical case for change’. At public meetings I observed local medical staff and members of the public confidently arguing against the plans, identifying weaknesses in the evidence for improvements in safety, suggesting alternative understandings and measures of ‘safety’, and drawing in other considerations, such as the distance patients would need to travel. By the end of fieldwork the plans had been abandoned due to community resistance.

Fifteen years later and global management experts, management knowledge, regions, and medical leaders, remain key elements of governing assemblages in healthcare. The following is from a news report (BBC news, 7 March 2013). The report is about a more recent campaign to save Lewisham Hospital in London. One march organised by the ‘Save Lewisham Hospital’ campaign was reported to have attracted 25,000 people (*The Times*, 27 Jan 2013). The Secretary of State for Health said that doctors had told him that the proposed changes would save 100 lives a year. In this case the community groups not only rejected the clinical framing, they self-organised to bring a judicial review which ruled in their favour (Cooper 2013). The following interaction, recorded at a public meeting, is between the Mayor of London, and a member of the public. I have included it here because it illustrates the way that medical expertise is mobilised in the politics of healthcare planning, and the inability of a politics of expertise to contain debate (Fischer 1990):

Mayor of London: The reforms that are proposed, just in respect of this area, could save in the region of 100 lives a year

Member of the public: Bollocks

## Knowledge artefacts

As the above analysis of hospital planning shows, in an ethnography of governing assemblages there are lots of ‘boring things’, such as power point slide packs, forms of analysis, and flowcharts. These knowledge artefacts do not just reflect underlying assumptions and ideas, they are important governing practices. For national policy actors they are powerful ways of shaping the activities of staff working in distant localities. They do this by defining, privileging, and constituting particular versions of healthcare. In this next section I use an example from my fieldwork to show how mundane practices of inscription and calculation standardise local activities in line with government objectives.

Between 2013 and 2015 I was doing fieldwork in four different parts of England as part of a government-funded evaluation of an integrated care programme. At this time layers of bureaucracy at the regional and local level had been removed and the functions of the central Department of Health had been dispersed to arms-length ‘partners’. The integrated care programme is an example of what is currently a dominant strand in healthcare policy in many countries –the promotion of partnerships between agencies and organisations to solve problems that are perceived to lie beyond the reach of any single organisation and can only be addressed if organisations work together in mutually beneficial ways (Bevir 2011). In this case national policy documents defined the overall objective – less fragmented care from the perspective of patients – but left the ‘how’ up to local networks (National Collaboration for Integrated Care and Support 2013). These networks were expected to work together to develop innovative service models. The local programmes were promised autonomy from national policy actors, although they were offered ‘support’ in the form of ‘coaching’, ‘tools’ and other resources (websites, webinars, workshops etc).

When I started fieldwork there were very different understandings of the policy between different local programmes (Jones 2017). One saw it as an ‘ethos’, that infused everything they did, another as a particular governing arrangement, namely a board that brought together relevant stakeholders, and another as ‘flexibilities’ and freedoms from national NHS managers. The subsequent plans were also diverse, and often radical. Many had long-term plans for preventing ill health by addressing ‘upstream’ factors that influence health and wellbeing, such as housing and early-years education. Most had goals that extended beyond health service outputs to include, for example, reductions in health inequalities, social inclusion, increased democratic participation, community capacity development, urban regeneration, and restorative justice. However, overtime, there was an observable reification of the policy as the activities of local programmes converged on a small number of concrete interventions, namely, a multi-disciplinary team and a care navigator (another example of an international model – see for example Bernstein and Razon 2019). There was also a convergence in the ‘programme theory’ as local programmes came to be conflated with discrete activities that would reduce costs by reducing emergency admissions to hospitals.

Although the formal bureaucratic structure of the NHS had been dismantled, hierarchical control continued in different forms. For example, funding for local plans was conditional on meeting targets for reducing emergency admissions to hospital. Organisations who failed to meet the target received a personal telephone call from the Health Secretary. Others received a personal visit from the NHS CEO (Jones 2017).

Alongside these visible forms of power, there were also less visible forms of control. Isomorphism is a feature of healthcare organisations (DiMaggio and Powell 1983), related to procedural legitimacy, and the circulation of management knowledge in the form of ‘models’, ‘tools’ and ‘techniques’ (Jones 2018). In this case, standardisation of organisational form was also accomplished through data collection and analysis, which worked as an indirect control technology to constitute a particular version of health care and channel the activities of local healthcare staff.

Although notionally autonomous, local programme managers were required to input data on the benefits of their plans into a form using pre-specified metrics (Table 1). The data was then collated in a ‘Data Pack’ – a Power Point slide pack – which was then shared with each local programme. The Data Pack presented the data that had been collected in graphs and tables to compare different localities on how they performed against the different metrics. In this way local programmes could view their own performance with other localities. However, the only way the form could be completed was to understand integrated care in a particular way, where the primary objective was to make cost savings. The form used to collect the data, and the graphs and tables used to present the analysis, encouraged local actors to understand the programme according to a shared language and logic (Rose and Miller 1992).

**Table 1.** Data template.

	Reduction in non-elective stays	Reduction in non-elective activity (%)	Saving (£m)	Reduction in delayed transfers of care (days)	Reduction in delayed transfers of care (%)	Saving (£m)	Reduction in permanent residential admissions (%)	Saving (£m)	Increase in reablement	Increased reablement (%)	Saving (£)	Other savings – from local metrics (£m)	Total savings (£m).
Name of locality													

The Department of Health referred to this process of data collection and analysis as ‘the stocktake’. ‘Taking stock’ as Foucault observed, is not just about examination but about correction (Foucault 1988). Whilst Foucault was referring to the individual, collective practices of data collection, analysis, and display act in a similar way. Localities are encouraged to compare themselves with others and correct. The effect was a standardisation of local activities. Gone were the radical experiments with restorative practice, and asset-based planning. And gone were local priorities for community cohesion and strengthening social support, to be replaced by a conception of integrated care as a means of saving money. The seemingly neutral act of data collection and analysis afforded the centre greater control over local activity through standardisation and alignment with central priorities for cost control. There were, however, instances of recognition, if not resistance. During a conversation with national NHS managers they said that the Stocktake had ‘a cool reception in some places’ with local actors concerned by the administrative burden and ‘feeling like they are being performance managed’.

One local programme stood out in the way it retained a focus on local objectives. A northern city, it had a strong geographical identity that emphasised local pride and political autonomy, and a history of ‘bottom up’ collaborative planning. In this case the lead organisation for the programme was the Local Authority. In England Local Authorities are responsible for a range of government services including social care, schools, housing and planning. In contrast to the top-down bureaucratic structure of the NHS, Local Authorities are made up of locally elected councillors who represent geographical wards and some of their funding comes from local taxes. At one of the board meetings, a national NHS manager attended, as a visitor, to share some data analysis. The analysis showed that in this locality general practitioners (family doctors) spent more money when compared to other localities. I watched as the chair, a local councillor, robustly challenged the authority of the NHS manager, and the relevance of the data analysis to local objectives. The chair told the NHS manager that they were spending more in some areas to address inequalities. Other members of the board also challenged the data analysis. One councillor said that the measures did not capture the concerns of her constituents, or the local programme’s strategic priorities. A patient representative said they did not include the patient experience. On three occasions during the board meeting members referred to the integrated care programme, maintaining that it gave them ‘flexibilities’ that they could use to enable their local strategy. In this instance the effects of a national policy were mediated by local political culture and shared ideas about the social determinants of health and wellbeing. The policy was also interpreted, and used, differently to other localities. The northern city retained their original interpretation of the policy, which was as an enabler of their local strategy, and they *used* the policy to legitimate and bolster their position.

## Conclusion

I have used ethnographic moments from the last fifteen years of fieldwork in the NHS in England to illustrate key features of healthcare governance: the way decisions are hidden in the places ‘in between’ and ‘out of reach’; the enrolment of doctors in governing; and the power of ‘boring things’ such as power point slides, flowcharts, and forms. These different elements interact, working together to align the activities of local healthcare managers and clinicians with the objectives of national policy actors. At the same time my fieldwork revealed a variegated discursive field and heterogenous effects as different policies interacted, with each other, and with the situated agency of local actors.

Comparing hospital planning with the integrated care programmes illuminates the different uses and effects of framing. In the case of hospital planning the framing was used as a rhetorical device, drawing on the cultural legitimacy of the medical profession. It was also a discursive strategy and political technology that recast hospital planning as technical issue, the domain of doctors, rather than a political issue open to public deliberation and decision-making. Other stakeholders, including local clinical staff and members of the community contested the framing. The framing has been, ultimately, ineffective. In the few cases where large-scale changes to clinical services have actually happened these required the coercive power of regional managers (Edwards and Buckingham 2020).

In the case of the integrated care programmes, the framing was also rhetorical, promising ‘person-centred care’, but it was also symbolic, of an ideological commitment to local autonomy and ‘less government’. This posed a problem for national governmental actors, in terms of how to accomplish their objective of major efficiency savings. To resolve this dilemma additional forms of power, both direct and indirect, were used to focus the activity of local managerial and clinical staff on this objective. The combination of different forms of power had an observable standardising effect in how the policy was enacted. However, one local programme rejected both hierarchical and governmental forms of control, using the policy framing to bolster their position. They maintained that the policy gave them ‘flexibilities’ to achieve their own locally defined objectives. In this way, the case of the integrated care programmes shows how policies are used by the governed, as well as those seeking to govern (Però 2011).

My ethnographic approach to governing assemblages has enabled me to link the discursive origins and properties of policy, to the intentions in, responses to, and effects of policies. While other studies have explored the discursive origins of policies, empirical research has focused on policy as presented in national policy documents, or in the interview accounts of policy elites. However, power does not operate apart from the way it shapes action. What anthropology contributes is an understanding of the influence of policy on practices, and a link to the tangible effects of policies. And while scholars have previously understood the operation of power as ‘assemblages’ of actors, knowledges, and practices, they have not explicated how these elements are brought together. I show how different discursive elements are brought into alignment through the agential act of framing. This is a novel approach that links agency, rhetoric, and discourse. My analysis complements existing scholarship where the focus is on the structuring and disciplining role of discourse. It does this, firstly, by showing the importance of agency in the operation of governmentality, and, secondly, by bringing to light the limits of governmentality. People contest and resist the frames of policy by mobilising alternative truths, suggesting, in turn, the need for more sovereign regimes of power to compensate for the failure of governmentality.

## References

[CIT0001] Abu, L. 1990. “The Romance of Resistance: Tracing Transformations of Power through Bedouin Women.” *American Ethnologist* 17 (1): 41–55. 10.1525/ae.1990.17.1.02a00030

[CIT0002] Ball, S. J. 1997. “Policy Sociology and Critical Social Research: A Personal Review of Recent Education Policy and Policy Research.” *British Educational Research Journal* 23 (3): 257–274. 10.1080/0141192970230302

[CIT0501] Bernstein, A., and N. Razon. 2019. “Anthropological Approaches to the Study of Health Policy.” *Human Organisation* 78 (1): 75–84. 10.17730/0018-7259.78.1.75

[CIT0003] Bevir, M. 2011. “Governance and Governmentality after Neoliberalism.” *Policy & Politics* 39 (4): 457–471. 10.1332/030557310X550141

[CIT0004] Brown, T. 2003. “Towards an Understanding of Local Protest: Hospital Closure and Community Resistance.” *Social & Cultural Geography* 4 (4): 489–506. 10.1080/1464936032000137920

[CIT0005] Burnham, P. 2001. “New Labour and the Politics of Depoliticisation.” *The British Journal of Politics and International Relations* 3 (2): 127–149. 10.1111/1467-856X.00054

[CIT0006] Carruthers, I. 2007. *Service Improvement: Quality Assurance of Major Changes to Service Provision*. London: Department of Health.

[CIT0007] Cooper, C. 2013. “Hunt lacked power to downgrade NHS services at Lewisham Hospital, Court of Appeal rules.” *Independent*, October 29. http://www.independent.co.uk/news/uk/politics/jeremy-hunt-lacked-power-to-downgrade-nhs-services-at-lewisham-hospital-court-of-appeal-rules-8911058.html

[CIT0008] Davidson, D., I. Williams, J. Glasby, and A. Ellis Paine. 2022. “Localism and Intimacy, and … Other Rather Imponderable Reasons of That Sort’: A Qualitative Study of Patient Experience of Community Hospitals in England.” *Health & Social Care in the Community* 30 (6): e6404–e6413. 10.1111/hsc.14083 36326043 PMC10092860

[CIT0009] Degeling, P. 1996. “Health Planning as Context‐Dependent Language Play.” *The International Journal of Health Planning and Management* 11 (2): 101–117. 10.1002/(SICI)1099-1751(199604)11:2<101::AID-HPM428>3.0.CO;2-Z 10159183

[CIT0502] DiMaggio, P., and W. Powell. 1983. “The Iron Cage Revisited: Institutional Isomorphism and Collective Rationality in Organizational Fields.” *American Sociological Review* 48 (2): 147–60. 10.2307/2095101

[CIT0503] Edelman, M. 1975. “Language, Myths and Rhetoric.” *Society* 12 (5): 14–2. 10.1007/BF02699914

[CIT0010] Edwards, N., and H. Buckingham. 2020. *Strategic Health Authorities and Regions: Lessons from History*. London: The Nuffield Trust.

[CIT0504] Ferlie, E. 2016. *Analysing Health Care Organisations: A Personal Anthology*. Abingdon: Routledge. 489–506. 10.1080/1464936032000137920

[CIT0011] Ferlie, E., and G. McGivern. 2014. “Bringing Anglo-Governmentality into Public Management Scholarship: The Case of Evidence-Based Medicine in UK Health Care.” *Journal of Public Administration Research and Theory* 24 (1): 59–83. 10.1093/jopart/mut002

[CIT0012] Fischer, F. 1990. *Technocracy and the Politics of Expertise*. Newbury Park: SAGE.

[CIT0013] Flinders, M., and M. Wood. 2014. “Depoliticisation, Governance and the State.” *Policy & Politics* 42 (2): 135–149. 10.1332/030557312X655873

[CIT0014] Foster, E., P. Kerr, and C. Byrne. 2014. “Rolling Back to Roll Forward: Depoliticisation and the Extension of Government.” *Policy & Politics* 42 (2): 225–241. 10.1332/030557312X655945

[CIT0015] Foucault, M. 1982. “Afterword. The Subject and Power.” In *Michel Foucault: Beyond Structuralism and Hermeneutics*, edited by P Dreyfus and M. Rabinow. Brighton: Harvester Press.

[CIT0016] Foucault, M. 1988. *Technologies of the Self: A Seminar with Michel Foucault*, edited by L. H. Martin, H. Gutman, and P. Hutton. Amherst: University of Massachusetts Press

[CIT0017] Foucault, M. 1990. “The History of Sexuality.” Volume 1: *An Introduction*. New York: Vintage.

[CIT0018] Foucault, M., G. Burchell, and C. Gordon. 1991. “Governmentality.” In *The Foucault Effect*, edited by P. Miller. Chicago: University of Chicago Press.

[CIT0505] Glasby, J., J. Smith, and H. Dickinson. 2006. *Creating ‘NHS Local’: A New Relationship between PCTs and local government*. Birmingham: University of Birmingham.

[CIT0019] Goffman, E. 1974. *Frame Analysis: An Essay on the Organization of Experience*. Harvard: Harvard University Press.

[CIT0020] Hay, C. 2014. “Depoliticisation as Process, Governance as Practice: What Did the ‘First Wave’ Get Wrong and Do we Need a ‘Second Wave’ to Put It Right?” *Policy & Politics* 42 (2): 293–311. 10.1332/030557314X13959960668217

[CIT0021] Ingold, T. 2014. “That’s Enough about Ethnography!.” *HAU: Journal of Ethnographic Theory* 4 (1): 383–395. 10.14318/hau4.1.021

[CIT0022] Jones, L. 2017. “Sedimented Governance in the English National Health Service.” In *Decentring Health Policy*, edited by M. Bevir and J. Waring, 17–33. London: Routledge.

[CIT0023] Jones, L. 2018. “Pastoral Power and the Promotion of Self‐Care.” *Sociology of Health & Illness* 40 (6): 988–1004. 10.1111/1467-9566.12736 29667210

[CIT0024] Jones, L., and M. Exworthy. 2015. “Framing in Policy Processes: A Case Study from Hospital Planning in the National Health Service in England.” *Social Science & Medicine (1982)* 124: 196–204. 10.1016/j.socscimed.2014.11.046 25461877

[CIT0200] Jones, L., and N. Fulop. 2021. “The role of professional elites in healthcare governance: Exploring the work of the medical director.” *Social Science & Medicine* 277: 113882. 33848720 10.1016/j.socscimed.2021.113882 PMC8135118

[CIT0026] Larner, W. 2003. “Neoliberalism?” *Environment and Planning D: Society and Space* 21 (5): 509–512. 10.1068/d2105ed

[CIT0027] Lynn, L. E. 2011. “The Persistence of Hierarchy.” *The SAGE Handbook of Governance*, edited by M. Bevir, 218–236. London: SAGE.

[CIT0028] March, J., and J. Olsen. 1989. *Institutions Rediscovered*. New York: Freedom Press.

[CIT0029] Marcus, G. E. 1995. “Ethnography in/of the World System: The Emergence of Multi-Sited Ethnography.” *Annual Review of Anthropology* 24 (1): 95–117. 10.1146/annurev.an.24.100195.000523

[CIT0030] McGivern, G., S. Dopson, E. Ferlie, M. Fischer, L. Fitzgerald, J. Ledger, and C. Bennett. 2018. “The Silent Politics of Temporal Work: A Case Study of a Management Consultancy Project to Redesign Public Health Care.” *Organization Studies* 39 (8): 1007–1030. 10.1177/0170840617708004

[CIT0031] McKinsey. 2009. *Achieving World Class Productivity in the NHS 2009-10 – 2013-14: Detailing the Size of the Opportunity*. London: McKinsey for the Department of Health.

[CIT0032] Miller, P., and N. Rose. 1993. “Governing Economic Life.” In *Foucault’s New Domains*, edited by M. Gane and T. Johnson. London: Routledge.

[CIT0507] Miller, P., and N. Rose. 2008. *Governing the Present: Administering Economic, Social and Personal Life*. Cambridge: Polity Press.

[CIT0506] National Collaboration for Integrated Care and Support. 2013. *Integrated Care and Support: Our Shared Commitment*. London: Department of Health.

[CIT0033] Però, D. 2011. “Introduction.” In *Policy Worlds: Anthropology and the Analysis of Contemporary Power*, edited by C. Shore, S. Wright, and D. Però. New York: Berghahn Books.

[CIT0034] Pushkar, P. 2023. “Accountabilities in the NHS: Coercion, Finance and Responsibility.” *The Cambridge Journal of Anthropology* 41 (1): 118–136. 10.3167/cja.2023.410109

[CIT0035] Rein, M., and D. Schön. 1993. “Reframing Policy Discourse.” In *The Argumentative Turn in Policy Analysis*, edited by F. Fischer and J. Forester. London: Duke University Press.

[CIT0036] Rhodes, R. 2013. “Political Anthropology and Civil Service Reform: Prospects and Limits.” *Policy & Politics* 41 (4): 481–496. 10.1332/030557312X655684

[CIT0037] Rose, N., and P. Miller. 1992. “Political Power beyond the State: Problematics of Government.” *The British Journal of Sociology* 43 (2): 173–205. 10.2307/591464 20092498

[CIT0038] Shore, C., S. Wright, and D. Però. 2011. *Policy Worlds: Anthropology and the Analysis of Contemporary Power*. New York: Berghahn Books.

[CIT0039] Star, S. L. 1999. “The Ethnography of Infrastructure.” *American Behavioral Scientist* 43 (3): 377–391. 10.1177/00027649921955326

[CIT0040] Stewart, E., S. L. Greer, A. Ercia, and P. Donnelly. 2020. “Transforming Health Care: The Policy and Politics of Service Reconfiguration in the UK’s Four Health Systems.” *Health Economics, Policy, and Law* 15 (3): 289–307. 10.1017/S1744133119000148 30975243 PMC7525102

[CIT0041] Waring, J. 2014. “Restratification, Hybridity and Professional Elites: Questions of Power, Identity, and Relational Contingency at the Points of Professional-Organisational Intersection.” *Sociology Compass* 8 (6): 688–704. 10.1111/soc4.12178

[CIT0042] Wedel, J. R. 2009. *Shadow Elite: How the World’s New Power Brokers Undermine Democracy, Government, and the Free Market*. Basic Books.

[CIT0043] Wright, S., and S. Reinhold. 2011. “‘Studying Through’: A Strategy for Studying Political Transformation.” In *Sex, Lies and British Politics. Policy Worlds: Anthropology and the Analysis of Contemporary Power*, 86–104. New York: Berghahn Books.

[CIT0044] Yanow, D. 1987. “Toward a Policy Culture Approach to Implementation.” *Review of Policy Research* 7 (1): 103–115. 10.1111/j.1541-1338.1987.tb00031.x

